# Attitudes of Canadian psychiatry residents if mentally ill: awareness, barriers to disclosure, and help-seeking preferences

**Published:** 2016-10-18

**Authors:** Tariq Hassan, Tanya Tran, Nam Doan, Mir Mazhar, Neeraj Bajaj, Tariq Munshi, Niall Galbraith, Dianne Groll

**Affiliations:** 1 Department of Psychiatry, Queen’s University; 2 Department of Psychology, Queen’s University; 3 Institute of Psychology, University of Wolverhampton, UK

## Abstract

**Background:**

The medical culture is defined by mental illness stigma, non-disclosure, and avoidance of professional treatment. Little research has explored attitudes and help-seeking behaviors of psychiatry trainees if they were to become mentally ill.

**Method:**

Psychiatry residents (*n* = 106) from training centres across Ontario, Canada completed a postal survey on their attitudes, barriers to disclosure, and help-seeking preferences in the context of hypothetically becoming mentally ill.

**Results:**

Thirty-three percent of respondents reported personal history of mental illness and the frequency of mental illness by year of training did not significantly differ. The most popular first contact for disclosure of mental illness was family and friends (*n* = 61, 57.5%). Frequent barriers to disclosure included career implications (*n* = *39*, 36.8%), stigma (*n* = 11, 10.4%), and professional standing (*n* = 15, 14.2%). Personal history of mental illness was the only factor associated with in-patient treatment choice, with those with history opting for more formal advice versus informal advice.

**Conclusions:**

At the level of residency training, psychiatrists are reporting barriers to disclosure and help-seeking if they were to experience mental illness. A majority of psychiatry residents would only disclose to informal supports. Those with a history of mental illness would prefer formal treatment services over informal services.

## Introduction

Physician well-being may be a lynchpin of good patient care. Poor physician physical and/or mental health can affect relationships with patients and impair their diagnoses and treatments, and the demanding nature of the job may compromise physician health and consequently patient care.^1^ A higher number of hospital call shifts was found to be associated with increased sleep deprivation and greater medical errors.^2^,^3^ Burnout and psychosocial stress have been linked with dampened patient-centered communication^4^ and lower self-assessed quality of care delivery.^5^ In the United States, physician licensing boards and credentialing bodies are increasingly asking physicians about a history of *severe* mental illness, citing that patient safety may be in question.^6^

Unfortunately, most physicians who develop a psychiatric illness that affects their personal, social, or occupational life tend to underreport and avoid appropriate treatment.^6^,^7^,^8^ In a Canadian study on the attitudes of practicing psychiatrists if they hypothetically developed mental illness, only 42% of psychiatrists indicated they would most likely disclose their mental illness to family and friends in the first instance, and stigma was cited as a factor influencing this choice.^9^ In a review on the mental health of doctors, the authors note, “It is important that doctors receive help quickly in order to protect the safety of their patients…”.^10^ As physicians are at a greater risk for depression, substance use disorders, and suicide, as compared to the general population,^11^ it is pertinent to investigate the help-seeking preferences of junior physicians if they were to become mentally ill.

A systematic review of research into depression, anxiety, and burnout in U.S. and Canadian medical students^12^ found 40 studies that suggested an overall prevalence of depression and anxiety among medical students that was greater than the general population and their age-matched peers. Amongst medical trainees, barriers to disclosure and help-seeking include perceived cultural expectations regarding self-reliance and not missing work,^13^ stigma associated with stress or mental illness, shame and embarrassment of admitting weakness, doubts that one’s confidentiality will be upheld upon disclosure to faculty or peers,^14^ and concerns related to one’s professional reputation.^15^ Moreover, perceived stigma of mental illness may seem to especially affect junior medical students as they more frequently endorse the opinion that help-seeking for depression would make them feel less intelligent.^16^ The concept of a “hidden curriculum” was reiterated in the revised 2014 report by the Canadian Federation of Medical Students (CFMS)^17^ This refers to the intentional process by which behaviours, skills, and knowledge are transmitted to learners by teachers, and the unintentional transmission of beliefs and ways of thinking and responding to individuals with mental illness that reinforces stigma surrounding mental illness, such as ridiculing patients.^17^ As reported in a London study, the hidden curriculum that perpetuates this form of stigma starts early on in training.^14^ As a result, junior medical students may be most vulnerable to perceived stigma of mental illness and, in turn, exercise non-disclosure.

The “Happy Docs Study”^18^ examined stress in Canadian interns and residents and found that 30% of residents reported having experienced a mental health problem with 23% reporting having had an emotional or mental health concern during their training. Compared to those who reported good mental health, significantly more of the residents who self-reported having poor or fair mental health indicated that they deal with stress by drinking alcohol.^18^ The “Happy Docs Study” also found that 14% of residents had consulted a psychiatrist or psychologist for help in the past, and that they would currently approach a resident colleague (65%), program director (53%), psychiatrist/psychologist (49%), chief resident (45%), or support telephone lines (33–44%) in a time of personal crisis.^18^ However, a study from the United Kingdom found that medical students tend to prefer to seek help from family and friends rather than formal services provided within or outside of the university.^14^ In one study surveying medical students from London, more than half of the students agreed it was appropriate for doctors to self-investigate and self-refer, and 40% even endorsed self-prescription. 19 This opinion was more frequently endorsed by senior medical students compared to more junior medical students.

A review of psychiatrists and non-psychiatry physicians and surgeons in Birmingham, UK found that having a pre-existing mental illness led to less stigma and a higher likelihood of seeking treatment quicker than those who would have presented with their first episode of a mental disorder.^20^ It is possible that initial disclosure to a loved one may, in itself, represent an important first step in seeking help from a mental health professional down the road.^21^ Research has not examined whether specialized training in psychiatry would affect attitudes, barriers to disclosure, and help-seeking if these professionals were to ever to develop a mental illness or the help-seeking preferences of those with a pre-existing mental disorder. Thus, this study used an exploratory approach to examine attitudes, barriers to disclosure, and help-seeking preferences of psychiatry residents if they hypothetically became mentally ill. Residents’ perceptions about their privacy as an in-patient may reflect on medical school or pre-clerkship experiences. Among the reasons identified by physicians for non-disclosure of illness was having “witnessed instances of failed confidentiality or obligatory reporting” in the past.^22^

Our hypotheses, based on the current literature, lead to the notion that junior doctors in training, who are not only vulnerable to mental illness, but are also susceptible to the stigma of mental illness, would probably shy away from effective help-seeking measures when faced with a first episode of mental illness. This may be in contrast to someone with prior experience of mental illness and help-seeking for a relapse in their condition. More junior trainees with less overall experience may have a less accepting attitude to mental illness than their senior counterparts and may perceive that mental illness in doctors is less prevalent than the general population. For the present study, the views of psychiatry residents, who are highly trained to carry out minimal judgment towards people with mental illness, were examined on their help-seeking preferences should they develop a first episode of a mental illness or a relapse of a current disorder.

## Methods

Prior to the commencement of this study, ethical approval was obtained from the Queen’s University and Affiliated Teaching Hospitals Research Ethics Board. This cross-sectional cohort study identified 301 psychiatry residents training in Ontario who were eligible to participate in the study, and their programs were mailed a survey package. Either the program administrator or the program director from each training site was tasked with distributing survey packages to residents. The package included a cover letter, a two-page questionnaire, and a stamped return addressed envelope. Consent was assumed based on participation in the survey. The surveys were sent out in January of 2014 and subsequently returned in April of 2014.

A ten-item questionnaire that had been previously used in studies of physicians’ attitudes towards mental illness was used in this study^9^,^19^,^20^ (Appendix 1). The questionnaire was comprised of three sections: the first collected information on the respondents’ perceptions of prevalence of mental illness in physicians compared to the general population, and psychiatrists compared to other medical/surgical professionals; the second required residents to identify to whom they would most likely disclose a mental illness and reasons for non-disclosure; the third section asked residents about their preference of treatment in both an out-patient and in-patient setting. Information on year of training and whether they had experienced mental illness in the past was also collected. Included in the survey were four screening questions for substance misuse in the last twelve months, answered in a Yes/No format. A free-form text box was included at the end for comments. Complete anonymity was maintained.

### Analysis

Responses from the paper questionnaires were entered into a Microsoft Excel spreadsheet designed for this study and then uploaded into SPSS. A series of two-sample chi-square *(X^2^)* tests were conducted to examine associations between categorical variables. In cases where 20% of contingency cells were < 5 or where any cell = 0, Fisher’s Exact test was used. Phi (*ϕ*) or Cramer’s *V* (for associations > 2×2) were used as measures of effect size providing an association coefficient between 0 and 1. All analyses were done using SPSS 22.

## Results

One hundred and six (35%) residents completed the survey. The respondents were classified by their year of training as PGY1 or PGY2 (*n* = 40, 37.7%), PGY3 or PGY4 (*n* = 49, 46.2%), or PGY5 or fellow (*n* = 17, 16%). The frequency of responses to all questions, both overall and as a function of these groups, are shown in Table 1.

### History of mental illness

Just over one third of respondents (*n* = 35, 33.0%) reported to have experienced a mental illness; there were no differences among PGY groups (χ^2^ = 1.06; *df* = 2; *p* = 0.588; Cramer’s *V* = 0.10).

### History of substance use

When asked about substance use in the last 12 months, only five respondents (4.7%) had smoked cigarettes, 37 (34.9%) had drank five or more drinks on one occasion, three (2.8%) had used sedatives, tranquilizers, or opioid analgesics, and ten (9.4%) had used illicit drugs. Due to the low numbers of those reporting substance use, the only relationship explored was between alcohol consumption and other variables, but no associations emerged (*p* ≥ 0.240).

### Perception of incidence of mental illness

Just under half of the respondents disagreed that the incidence of mental illness was higher in doctors compared to the general population (*n* = 50, 47.2%). A smaller, but sizeable, proportion agreed with that statement (*n* = 40, 38.7%), while some replied “don’t know” (*n* = 15, 14.2%). As can be seen in Table 1, the pattern of response was similar across all PGY groups for this question (*χ^2^* = 1.58; *df* = 4; *p* = 0.819; Cramer’s *V* = 0.09). Most disagreed that psychiatric illness was greater in medical/surgical professionals than in psychiatrists (*n* = 63, 59.4%), a small minority agreed (*n* = 17, 16.0%). Again, the PGY groups responded similarly for this question (*χ^2^* = 5.62; *df* = 4; *p* = 0.229; Cramer’s *V* = 0.16).

### Disclosure of mental illness

Respondents would be most likely to disclose their first instance of mental illness to family and friends (*n* = 61, 57.5%), although many would instead prefer to disclose to their GP/family physician (*n* = 30, 28.3%). Relatively few respondents would disclose to a colleague (*n* = 5, 4.7%), very few would choose noone (*n* = 3, 2.8%), and the clergy was the least endorsed option (*n* = 1, 0.9%). When considering only the two most popular response options (family/friends, GP/family physician), there were no significant differences among the three PGY groups (*χ^2^* = 1.99; *df* = 2; *p* = 0.370; Cramer’s *V* = 0.15).

When asked about the most important factor affecting the decision not to disclose, the most common response was career implications (*n* = 39, 36.8%); however, stigma (*n* = 11, 10.4%) and professional standing (*n* = 15, 14.2%) were also offered by a minority of respondents.

### Treatment for mental illness

When considering out-patient treatment, the majority of respondents would opt for formal professional advice (*n* = 73, 68.9%). A smaller proportion would choose informal professional advice (21, 19.8%) and very few would self-medicate (*n* = 6, 5.7%) or choose no treatment (*n* = 2, 1.9%). With regard to in-patient treatment, the majority would opt for an out of area mental health facility (*n* = 88, 83.0%). Only a small minority of respondents (*n* = 8, 7.5%) reported that quality of care would influence their choice of in-patient care, while nearly two thirds would be most concerned about confidentiality (*n* = 67, 63.2%). Some cited convenience (*n* = 11, 10.4%) and some cited stigma (*n* = 15, 14.2%) as factors in the decision. There was a strong association between in-patient preference and the factor influencing that preference (Fisher’s Exact = 40.72; *p* < 0.001; Cramer’s *V* = 0.73). As shown in Table 2, those who would choose an out of area facility were much more likely to cite confidentiality and stigma as factors influencing their choice, compared to those who would choose a local facility. Conversely, those choosing a local facility were more likely to cite quality of care and convenience as influencing factors. There was no association between PGY group and out-patient preference, in-patient preference, nor factors influencing in-patient choice *(p*-values > *0.05*).

Finally, previous experience of mental illness was not associated with out-patient choice (*χ^2^* = 6.20; *df* = 3; *p* = 0.102; Cramer’s *V* = −0.25), but it was associated with in-patient choice (*Fisher’s Exact* p *= 0.001; ϕ = .34*). As Table 3 indicates individuals who had not experienced mental illness were more likely to prefer out of area in-patient treatment than those, who had previously experienced mental illness.

## Discussion

This is the first study to ask medical residents what their response would be if they developed a mental illness, who they would turn to for help, if they would disclose their illness and to whom, and why they would or would not disclose. Overall, this study found that approximately one-third of responding psychiatry residents had a personal history of mental illness, similar to the proportion reported in previous studies,^7^–^9^ and the percentage of personal history of mental illness in our population did not differ significantly by year of training. Prior to the initiation of this study, we hypothesized that with more years of training, psychiatry residents would have more accepting attitudes of mental illness, they would more likely disclose their mental illness to formal supports, and they would prefer more formal help for an illness compared to more junior trainees. However, we found that higher year of training was not related to more accepting attitudes of mental illness. Nearly half of our sample of psychiatry residents disagreed that the incidence of mental illness was higher in doctors than the general population, when in fact the literature suggests otherwise^11^; this opinion did not differ by year of training. Regardless of the amount of experience psychiatry residents may have, a large portion seem not to be aware of the prevalence of mental illness in their medical community.

Moreover, consulting a friend or family member first if they were to become mentally ill was the most common option chosen by respondents. This is in line with previous research on practicing Ontario physicians who also preferred a family member or friend as a first point of contact for disclosure of their mental illness.^9^ Contrary to our hypothesis, level of training was not associated with choice of formal and informal contacts to whom they would disclose their mental illness. Findings suggest a reluctance in the medical trainee community, regardless of year of training, to disclose mental health problems to professionals. However, we also found that 97.2% of respondents would choose disclosure of their mental illness as opposed to not, irrespective of whether the first point of contact would be a professional or a personal acquaintance.

Important barriers to disclosure in our sample of psychiatry residents were career implications, professional standing, and stigma. These findings are consistent with a previous survey of physicians in Ontario^9^ as well as results from studies surveying medical students and practicing physicians internationally.^11^,^13^,^19^–^21^ More psychiatry residents were concerned about confidentiality in their choice of location for in-patient care (local vs. out of area), rather than quality of care. Year of training was also not associated with help-seeking preferences, as hypothesized; instead, personal history of mental illness was associated with choice of in-patient service. This reflects previous research on Ontario physicians^9^ that found, while all respondents cited confidentiality as their primary reason for choice of in-patient care, older physicians (those with ten or more years of practice) were significantly more likely to choose quality of care as their reason for choice of in-patient location than junior physicians (< 5 years of practice), who were significantly more likely to cite confidentiality as their primary concern.

Psychiatry residents who had experienced mental illness were more likely to opt for informal outpatient services over formal in-patient services, as compared to those without history of mental illness. It may be that psychiatry residents who have personal experience with mental illness are distrustful of formal services. In light of these findings, and other research that found senior medical trainees endorsed self-investigation or self-medication as an appropriate treatment for their mental illness,^19^ more research into the concerns surrounding confidentiality or stigma associated with formal services, and how they can be addressed, needs to be conducted.

Concerns of confidentiality and career implications in relation to disclosure and help-seeking preferences may be as equally reflective of valid objective concerns as they are of the residents’ subjective fears. The relative similarity of responses in our sample across resident seniority levels may indeed point to the importance of pre-residency influences.

In 2010, the Canadian Medical Association (CMA) published a physician’s mental health strategy paper to address the burden of mental health issues and illness in trainees and practicing physicians, including, what they described as, the culture and stigma surrounding mental illness, as well as confidentiality and licensing concerns.^23^ Four strategic directions were outlined; the third pertained to creating learning and work environments that support the mental health of physicians. Within the third strategic direction, medical schools and graduate programs were tasked to: “foster networking and mutual support among students and residents; address stigma within the training environment and eliminate the ‘hidden curriculum’; support positive learning experiences; support a continuum of programs and services to address mental health issues and illness, including healthy approaches to resilience and coping”.^23^ As reported in a London study, the “hidden curriculum” that perpetuates this form of stigma starts early on in training.^14^ Addressing the mental health of trainees and confronting the attitudes and beliefs that may result in the perpetuation of stigma, thereby affecting disclosure of mental health issues, are the focuses of the CFMS report. The CFMS report’s four recommendations for medical schools are to:^23^

1. Develop formal and informal mental health and wellness support initiatives at each medical school in Canada.
2. Develop mental health awareness initiatives at both the university and national level.
3. Support research exploring mental health in medical learners and evaluating mental health and wellness initiatives.
4. Establish accessible and realizable standards for accommodation of medical students with mental illnesses in each university’s policies for each phase of undergraduate medical training.

It is the responsibility of the medical school to operationalize these recommendations in order to maximize the mental health potential of their medical trainees, and create an environment that supports mental wellness.

There are several important limitations of this study that need to be considered. This survey was carried out in only one Canadian province, therefore the generalizability of the results outside of Ontario cannot be assumed. The response rate of residents to this survey was 35%. While this response rate is similar to other surveys of Canadian physicians and trainees,^18^,^24^ and twice that of the National Physician Survey of Medical Residents,^25^ responses may not represent the views of Ontario psychiatry residents. Our interpretation of the data is provisional in light of the response rate; however, the low response rate may also be reflective of the hesitancy to disclose attitudes and experiences with mental illness within the medical community. As with any study that asks an individual to speculate as to what they would do under a given circumstance, the responses may or may not reflect what they would actually do in that circumstance. It is also possible the responses in this study reflect what the residents believe are professionally appropriate answers. Nevertheless, there is scope for more research in this area, whether in terms of psychiatry residents in other training centres, or exploring the psychosocial antecedents of mental health stigma in the medical community.

### Conclusion

This is the first study to examine residents’ intended disclosure of their own mental illness if it developed, and their subsequent help-seeking preferences. Our hypotheses that the junior doctors would probably shy away from effective help seeking measures when faced with a first episode of mental illness, and that the more junior trainees with overall less experience may perceive that mental illness in doctors is less prevalent than the general population, were not supported by our data. There were no statistically significant differences between the PGY groups for any of our areas of inquiry. These findings are similar to those of non-trainee physicians.^9^,^20^

The CMA strategy paper^22^ has a list of recommendations for the future of physician health care in Canada. Many of these recommendations are preventative measures targeting medical students and residents. The recommendations we believe to be the most relevant with respect to this study are: Recommendation III - Personal physician for every physician, whereby physicians have a family physician trained to treat physicians who otherwise may self-medicate and resist formal help; and Recommendation IV - Engaging the regulatory bodies, who need to address their roles in perpetuating stigma, and differentiate between illness and impairment to ensure physicians with mental health issues do not feel that they cannot disclose without ending or damaging their career.

The results of this study suggest that stigma for mental health disclosure exists even for those studying psychiatry. Research has shown that stigma continues to be prevalent in physicians and physicians-in-training as it pertains to the attitudes, disclosure, and help-seeking preferences for mental illness. We suggest future research focus on further identification of the systemic factors that reinforce and perpetuate stigma in the medical community.

We consider this to be a compelling resource giving direction for future research on student, resident, and attending physician wellbeing.

## Figures and Tables

**Table 1:** Frequency statistics for all questions divided by PGY group

Question		PGY Group			
		Overall	PGY1–2	PGY3–4	PGY5/Fellow
Incidence of psychiatric illness amongst doctors is higher than general population?	No	50 (47.2%)	18 (45.0%)	24 (49.0%)	8 (47.1%)
	Yes	41 (38.7%)	15 (37.5%)	18 (36.7%)	8 (47.1%)
	Don’t know	15 (14.2%)	7 (17.5%)	7 (14.3%)	1 (5.9%)

Incidence of psychiatric illness amongst medical/surgical professionals higher than that of psychiatrists?	No	63 (59.4%)	23 (57.5%)	27 (55.%)	13 (76.5%)
	Yes	17 (16.0%)	4 (10.0%)	11 (22.4%)	2 (11.8%)
	Don’t know	26 (24.5%)	13 (32.5%)	11 (22.4%)	2 (11.8%)

Have you ever experienced a mental illness, which had affected your personal, social or occupational life?	No	68 (64.2%)	25 (62.5%)	30 (61.2%)	13 (76.5%)
	Yes	35 (33.0%)	15 (37.5%)	16 (32.7%)	4 (23.5%)

In the last 12 months did you use…	Cigarettes	5 (4.7%)	4 (10.0%)	1 (2.0%)	0 (0.0%)
	Alcohol	37 (34.9%)	16 (40.1%)	16 (32.7%)	5 (29.4%)
	Sedatives	3 (2.8%)	3 (7.5%)	0 (0.0%)	0 (0.0%)
	Illicit drugs	10 (9.4%)	6 (15.0%)	4 (8.2%)	0 (0.0%)

If you were to develop a psychiatric illness affecting your personal, social or occupational life, to whom would you initially be most likely to disclose this?	Church/Clergy	1 (0.9%)	0 (0.0%)	0 (0.0%)	1 (5.9%)
	GP/Family Physician	30 (28.3%)	9 (22.5%)	17 (34.7%)	4 (23.5%)
	Family/Friends	61 (57.5%)	25 (62.5%)	25 (51.0%)	11 (64.7%)
	Colleagues	5 (4.7%)	2 (5.0%)	3 (6.1%)	0 (0.0%)
	No-one	3 (2.8%)	1 (2.5%)	1 (2.0%)	1 (5.9%)
	Other	6 (5.7%)	3 (7.5%)	3 (6.1%)	0(0.0%)

What is the most important factor that would affect your decision not to disclose your mental illness?	Stigma	11 (10.4%)	5 (12.5%)	5 (10.2%)	1 (5.9%)
	Career Implications	39 (36.8%)	18 (45.0%)	14 (28.6%)	7 (41.2%)
	Professional Standing	15 (14.2%)	6 (15.0%)	8 (16.3%)	1 (5.9%)
	Other	5 (4.7%)	3 (7.5%)	1 (2.0%)	1 (5.9%)

If you were to suffer from a mental illness affecting your personal, social or occupational life requiring out-patient treatment, what would be your first treatment preference?	Informal Profess. Advice	21 (19.8%)	9 (22.5%)	9 (18.4%)	3 (17.6%)
	Formal Profess. Advice	73 (68.9%)	29 (72.5%)	32 (65.3%)	12 (70.6%)
	Self-medication	6 (5.7%)	1 (2.5%)	3 (6.1%)	2 (11.8%)
	No Treatment	2 (1.9%)	1 (2.5%)	1 (2.0%)	0 (0.0%)

If you were to develop a mental illness requiring **in-patient** treatment, where would be your **first** preference?	Local	15 (14.2%)	8 (20.0%)	6 (12.2%)	1 (5.9%)
	Out of Area	88 (83.0%)	32 (80.0%)	40 (81.6%)	16 (94.1%)

In choosing in-patient preference, which of the following influenced your decision **most**?	Quality of care	8 (7.5%)	3 (7.5%)	4 (8.2%)	1 (5.9%)
	Convenience	11 (10.4%)	8 (20.0%)	2 (4.1%)	1 (5.9%)
	Confidentiality	67 (63.2%)	25 (62.5%)	32 (65.3%)	10 (58.8%)
	Stigma	15 (14.2%)	4 (10.0%)	8 (16.3%)	3 (17.6%)

**Table 2:** Frequency statistics of in-patient treatment choice and the factors influencing that choice

In-patient treatment choice	Factors influencing in-patient choice				
	Quality of Care	Convenience	Confidentiality	Stigma	Total *n*

**Local**	6 (40.0%)	7 (46.7%)	2 (13.3%)	0 (0.0%)	15

**Out-of-area**	2 (2.3%)	4 (4.7%)	65 (75.6%)	15 (17.4%)	86

**Table 3:** Frequency statistics of personal history of mental illness and in-patient treatment choice

In-patient treatment choice	Personal history of mental illness		
	No	Yes	Total n

**Local**	4 (26.7%)	11(73.3%)	15

**Out-of-area**	64 (72.7%)	24 (27.3%)	88

## Appendix 1

### Survey of Psychiatry Residents Attitudes to Becoming Mentally Ill

1. Are you a: PGY 1 or PGY 2
  PGY 3 or PGY4
  PGY 5 or Fellow
2. Do you think that the incidence of psychiatric illness amongst doctors is **higher** than that of the **general population**? (*Please circle one*)
  Yes No Don’t know
3. Do you think that the incidence of psychiatric illness amongst medical/surgical professionals is **higher** than that of **psychiatrists**? (*Please circle one*)
  Yes No Don’t know
4. In the last 12 months (Circle Yes or No):
  - Did you smoke cigarettes on a regular basis? Yes No
  - Did you have 5 or more drinks of alcohol in the same occasion? Yes No
  - Did you use sedatives, tranquilizers or opioid analgesics without a prescription? Yes No
  - Did you use any illicit drugs such as cannabis, cocaine, methamphetamine, etc.? Yes No
5. If you were to develop a psychiatric illness affecting your personal, social or occupational life, to whom would you initially be **most** likely to disclose this? (Please check **one only**)
  Church Clergy
  General Practitioner/Family Physician
  Family & Friends
  Colleagues
  None
  Other (please specify):_______________
6. What is the **most** important factor that would affect your decision not to disclose your mental illness? (Please check **one only)**
  Stigma
  Career Implications
  Professional standing
  Other (please specify):_______________
7. If you were to suffer from a mental illness affecting your personal, social or occupational life requiring **out-patient** treatment, what would be your **first** treatment preference? (*Please check* ***one only***)
  - Informal professional advice
  - Formal professional advice
  - Self medication
  - No treatment
8. If you were to develop a mental illness requiring **in-patient** treatment, where would be your **first** preference? (*Please check* ***one only***)
  - Local Mental Health Facility
  - Out of area Mental Health Facility
9. In choosing the place of treatment in Question 8, which of the following influenced your decision **most**? (*Please circle* ***one only)***
  Quality of care
  Convenience
  Confidentiality
  Stigma
  Other (please specify): _______________
10. Have you ever experienced a mental illness, which had affected your personal, social or occupational life? (Please circle one)
  Yes No

Additional Comments:

_____________________________________________________________________________________________

_____________________________________________________________________________________________

_____________________________________________________________________________________________

Thank you for your participation.
